# Potential Lung Nodules Identification for Characterization by Variable Multistep Threshold and Shape Indices from CT Images

**DOI:** 10.1155/2014/241647

**Published:** 2014-11-25

**Authors:** Saleem Iqbal, Khalid Iqbal, Fahim Arif, Arslan Shaukat, Aasia Khanum

**Affiliations:** ^1^CEME, National University of Science and Technology, Islamabad 46000, Pakistan; ^2^MCS, National University of Science and Technology, Islamabad 46000, Pakistan

## Abstract

Computed tomography (CT) is an important imaging modality. Physicians, surgeons, and oncologists prefer CT scan for diagnosis of lung cancer. However, some nodules are missed in CT scan. Computer aided diagnosis methods are useful for radiologists for detection of these nodules and early diagnosis of lung cancer. Early detection of malignant nodule is helpful for treatment. Computer aided diagnosis of lung cancer involves lung segmentation, potential nodules identification, features extraction from the potential nodules, and classification of the nodules. In this paper, we are presenting an automatic method for detection and segmentation of lung nodules from CT scan for subsequent features extraction and classification. Contribution of the work is the detection and segmentation of small sized nodules, low and high contrast nodules, nodules attached with vasculature, nodules attached to pleura membrane, and nodules in close vicinity of the diaphragm and lung wall in one-go. The particular techniques of the method are multistep threshold for the nodule detection and shape index threshold for false positive reduction. We used 60 CT scans of “Lung Image Database Consortium-Image Database Resource Initiative” taken by GE medical systems LightSpeed16 scanner as dataset and correctly detected 92% nodules. The results are reproducible.

## 1. Introduction

Lungs are important organs of the body for survival of human beings. The lungs may suffer from many fatal diseases. Lung cancer is the most fatal of all these diseases. It is a leading cause of cancer related deaths in the world. Early diagnosis of lung cancer is a must for success of therapy. Imaging modalities play an important role in diagnosis of lung cancer. Positron emission tomography (PET) and computed tomography (CT) are the most common noninvasive modalities for detection and diagnosis of lung cancer. PET scan is well known for discrimination of malignant and benign nodules. CT technology is helpful for early detection of malignant nodules. CT technology for diagnosis of lung cancer is entering into its new phase known as computer aided diagnosis (CAD).

CAD systems are active area of research of the day. Automated CAD systems are useful for radiologists for detection and diagnose of lung cancer from CT slices. A CAD system is made up of subsystems. Important ingredients of a CAD system are lung segmentation subsystem, potential pulmonary nodule identification (PNI) subsystem, features generation subsystem and nodules classification subsystem. Potential PNI subsystem is the key component of a CAD system. A nodule overlooked during pulmonary nodule identification phase is always missed for the rest of the process. The present research is focused on PNI.

PNI is a prerequisite for diagnostic and treatment procedures for lung cancer [1]. It is used in the CAD systems for diagnosing of tumor growth in successive computed tomography scans [2, 3] and monitoring therapy success [4, 5], lung cancer screening for early detection by computer aided methods [6–8], and computer aided diagnosis of malignancy of lung cancer [9, 10].

PNI involves nodules detection and nodules segmentation. Lung nodule segmentation is the delineation of the nodular lesions appearing on the lung CT. Nodule detection is the recognition of an object as a nodule. The nodule segmentation and detection are important and crucial steps in lung cancer diagnosis applications. Some techniques have been proposed and utilized by the researchers for PNI. Thresholding [11, 12], region growing [13, 14], dynamic programming [15], mean shift [16], watershed transform [17], mathematical morphology [18, 19], deformable model [20, 21], and model fitting [22, 23] are the techniques reported by the scientists and engineers for PNI.

These works [11–23] mainly focused on features extraction and classification for PNI. Features extraction and classification are time consuming and costly processes, particularly for large number of CT slice images (240 to 300 images) in a single CT scan. Our objective is accurate identification of potential pulmonary nodules without features extraction and classification.

**Figure 1:**
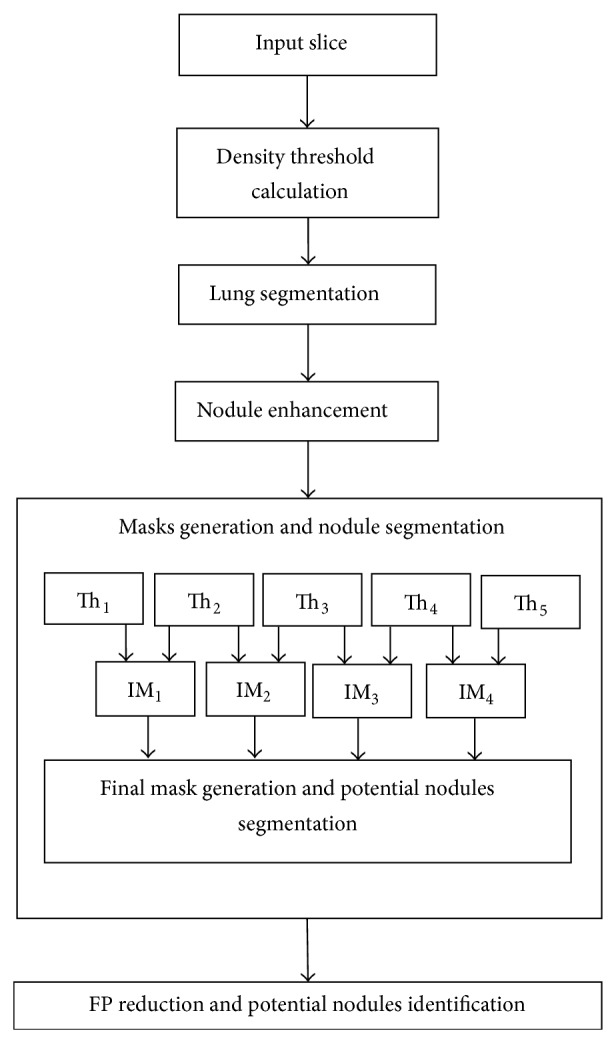
Flow diagram. IM stands for intermediate mask. Th stands for threshold (intermediate threshold). FP stands for false positives.

The proposed system takes a CT slice as input, calculates density threshold value, performs lung segmentation, enhances the nodular object on image, generates nodule masks, carries out nodule segmentation, and reduces the number of false positives. The process is performed for each slice of the CT scan. The flow diagram of the system is shown in Figure 1.

## 2. Material and Method

In the lung CT scan, nodules are objects comparatively brighter than the background with spherical/elliptical shape or having embedded spherical/elliptical objects upon them besides their particular visual properties and pathologies. Figure 1 gives a general idea of the proposed lung nodule detection system. The following sections illustrate material and methods.

Section 2.1 describes the dataset.  Section 2.2 illustrates calculation of density threshold which was subsequently used for lung segmentation and nodules identification process.  Section 2.3 describes lung segmentation. Segmented lung images are input for nodule identification process.  Section 2.4 mentions lung enhancement.  Section 2.5 explains nodule masks generation and candidate nodule segmentation processes.  Section 2.6 describes false positive reduction process.

### 2.1. Data Set

LIDC-IDRI (Lung Image Database Consortium-Image Database Resource Initiative) [24] is a globally accessible resource for development and testing of CAD methods for lung cancer detection and diagnosis. We used available 60 CT scans taken by GE medical systems LightSpeed16 scanner. The total number of slices/images is 8573. There are 222 nodules in all. The size of the nodules is 3–30 mm (millimeters). All the 60 scans contain nodules. However, there are a large number of slices in these scans which do not contain modules. Each slice has 12 bit gray scale resolution.

### 2.2. Density Threshold Calculation

We calculated density threshold value for lung segmentation and nodules identification process by Algorithm 1.

Value 0.9 is experimental value showing better segmentation results.

Algorithm 1 takes a slice of the CT scan as input and gives as an output a density value. Step (1) calculates mean of all the density values of the input slice and names it as “IT.” “IT” is the initial threshold value. The slice (image) is thresholded using the value “IT.” Two clusters of density values are formed. As stated in Steps (2) and (3), mean of each cluster is calculated. According to Step (4), mean of means of the two clusters is computed and named as “T.” “T” is the new threshold value. In Step (5), two threshold values “IT” and “T” are compared. If difference of “IT” and “T” is greater than 0.9, new threshold value “T” becomes initial threshold “IT” and Step (2) to Step (5) are repeated. If the difference of “IT” and “T” is less than or equal to 0.9, “T” is the final threshold value. The utilization of this threshold value is in Lung Segmentation (Section 2.3) and in Five Threshold Value Calculation (Section 2.5.1).

### 2.3. Lung Segmentation

**Figure 2:**
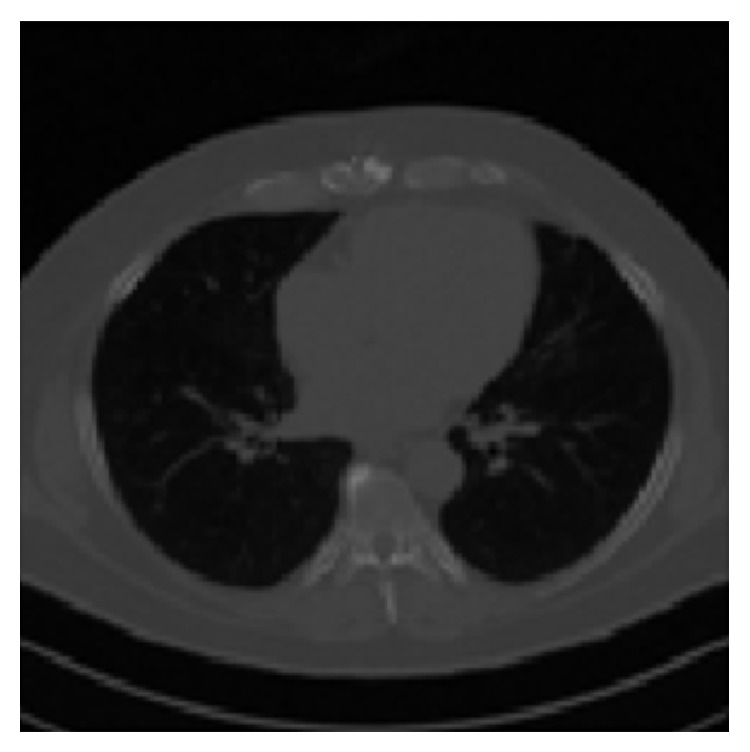
CT slice image.

Lung segmentation is a prerequisite for computer aided lung cancer diagnosis system. CT slice image of the lung is shown in Figure 2. We segmented the lung regions from CT slice using density threshold, mathematical morphology, and pixel connectivity.

Lung regions were approximated by thesholding, using a threshold value calculated in Section 2.2. There are four problems to be solved on the approximated lungs. There are boundary connected objects other than lungs on slice images. There are some missing portions (gaps) on the lung regions. Trachea exists which is not part of the lung. Boundaries of lung regions are required to be corrected. Resolution of these problems leads to final segmented lungs.

**Figure 3:**
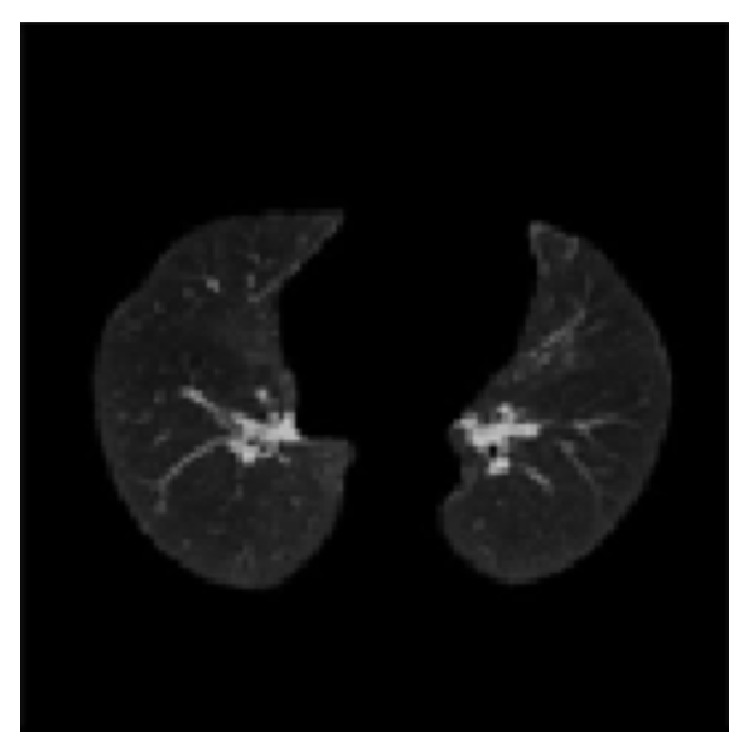
Segmented lung image.

We refined lung in previous works [30, 31]. In the works, we removed objects connected with the border of the CT slice image using pixel connectivity concept. We recognized all the objects on the slice and removed those objects which have at least one common pixel with the boundary of the slice. These objects were due to attenuation of X-rays through the air around the patient, partial volume effect, and different artifacts. Gaps on the region of interest were stuffed by Flood Fill Algorithm (FFA) on binary lung mask. The FFA fills closed gaps on the objects. Trachea was removed by exploiting anatomical property. Anatomically, trachea is smaller in size than left and right lungs. We compared the size of trachea with left and right lungs and removed trachea (smaller object in size). We corrected lung boundaries by mathematical morphology. For resolution of poor demarcation along the boundaries of the lungs, the boundaries of the mask were smoothed by morphological closing. Firstly, structuring element of size one was tried but it could not cover entire portion of the lung. More accurate segmentation resulted with structuring element of size 2. Structuring element of size 3 covered some portion of chest body along with lungs. Resultant final segmented lungs are shown in Figure 3.

### 2.4. Nodule Enhancement

Image enhancement brings out and highlights some specific features, characteristics, and objects of the image. It denoises and smoothes out the image. It is helpful for nodules discrimination from adjacent anatomical structures. Manay and Yezzi [32] proposed anti-geometric diffusion model for denosing and smoothing. We have utilized the model for nodule enhancement. Good thing about the model is that it diffuses edges of the image. The advantages of the diffusion across the edges include better localization, better connectivity of the shape index map, and less noise sensitivity. According to the model, if “*g*” and “*t*,” respectively, denote the gradient and tangent direction of the iso-intensity contour of an image “*I*,” then anti-geometric diffusion is defined as

(1) ∂I∂t=Igg  or∂I∂t=I2xIxx+2IxIyIxy+I2yIyyI2x+I2y.

*I* ^2^ *x*, *I*_*xx*_, *I*_*x*_, *I*_*y*_, *I*_*xy*_, and *I* ^2^ *y* denote derivatives of image in usual notations.

Nodule enhancement proved particularly useful in recognition of low density nodules from the central parts of the parenchyma of left and right lung.

### 2.5. Masks Generation and Nodule Segmentation

We calculated five threshold values and developed four intermediate masks. We added four intermediate binary masks using the principal of addition of images to generate the final binary mask for nodule identification. The final binary mask identifies nodules on the original slice images. On the binary mask, nodule regions have the intensity values as “one” and the rest of lung region have the intensity values as zero. The procedure is described below in more detail.

#### 2.5.1. Five Threshold Values Calculation

The threshold value calculated in Section 2.2 is the “base threshold” for five threshold values calculation. The “maximum intensity value” of the slice is divided by 10 to make a “step value.” “Step value” is a numerical value which is added to or subtracted from the base value in order to calculate more threshold values. The first threshold value, Th_1_ is the base threshold value plus two times step value. Second threshold value, Th_2_, is the base threshold value plus step value. Third threshold value, Th_3_, is the base value itself. Fourth threshold value, Th_4_, is the base threshold value minus the step value. Fifth threshold value, Th_5_, is the base threshold value minus two times step value.

#### 2.5.2. Intermediate Mask Generation

Four intermediate masks IM_1_, IM_2_, IM_3_, and IM_4_ are generated by using the threshold values Th_1_, Th_2_, Th_3_, Th_4_, and Th_5_. The intermediate mask IM_1_ is generated using Th_1_ and Th_2_. For generation of intermediate mask IM_1_, on the original CT slice image, intensity values less than or equal to the threshold value Th_1_ and greater than Th_2_ are assigned values as “1” and intensity values greater than Th_1_ and less than or equal to Th_2_ are assigned values equal to “0.” We improved the mask IM_1_ by morphological opening operation with disk as a structuring element, 2 being the size of the structuring element. Using a similar procedure, intermediate masks, IM_2_, IM_3_, and IM_4_ were developed using threshold values Th_2_ and Th_3_, Th_3_ and Th_4_, and Th_4_ and Th_5_, respectively. It is worth mentioning that Th_1_ > Th_2_ > Th_3_ > Th_4_ > Th_5_.

#### 2.5.3. Final Mask Generation and Candidate Nodules Segmentation

We generate a single final binary mask, NC_Mask, from the four intermediate masks IM_1_, IM_2_, IM_3_, and IM_4_ utilizing image addition concept. Mathematically,

(2) $$\begin{array}{c} \text{NC}\_\text{Mask}\left(i,j\right)=\overset{4}{\underset{k=1}{\sum }}{\text{IM}}_{k}\left(i,j\right). \end{array}$$

NC_Mask(*i*, *j*) is the final single mask for PNI. The single mask for nodule identification saves a lot of computation and avoids complexity as compared to the process of nodule detection from four different binary masks and then showing the resultant nodule objects.

Objects on binary nodule mask reveal the nodule objects on original CT slice images. We segmented PNI from original CT slice images by array multiplication of original CT images and respective binary nodule masks.

### 2.6. False Positive Reduction and Potential Nodules Identification

Shape index indicates the geometrical nature of the objects on the image. Different shapes have different shape index values [33]. The “cup,” “rut,” “saddle,” “ridge,” and “cap” are important shape classes in image objects. The shape index values for the shapes “cup,” “rut,” “saddle,” “ridge,” and “cap” are 0.00, 0.25, 0.50, 0.75, and 1.0, respectively [33]. Shape index map of an image is a map of shape indices of the image. Shape index (SI) of a voxel *V*_*ij*_ is calculated as

(3) $$\begin{array}{c} \text{SI}\left(V_{ij}\right)=\frac{1}{2}-\frac{1}{\pi }\tan^{-1}\frac{K1\left(V_{ij}\right)+K2\left(V_{ij}\right)}{K1\left(V_{ij}\right)-K2\left(V_{ij}\right)}. \end{array}$$

**Figure 4:**
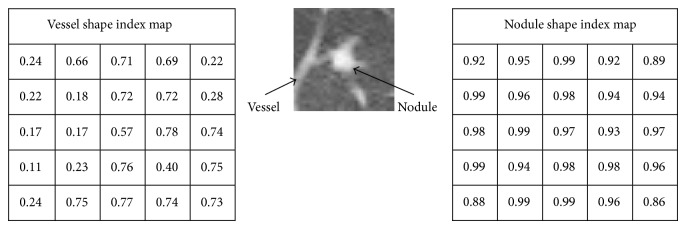
Shape index of nodule and vessel.

*K*1(*V*_*ij*_) and *K*2(*V*_*ij*_) are principal curvature of voxel, *V*_*ij*_. Shape index of a vessel and nodule are shown in Figure 4.

**Figure 5:**
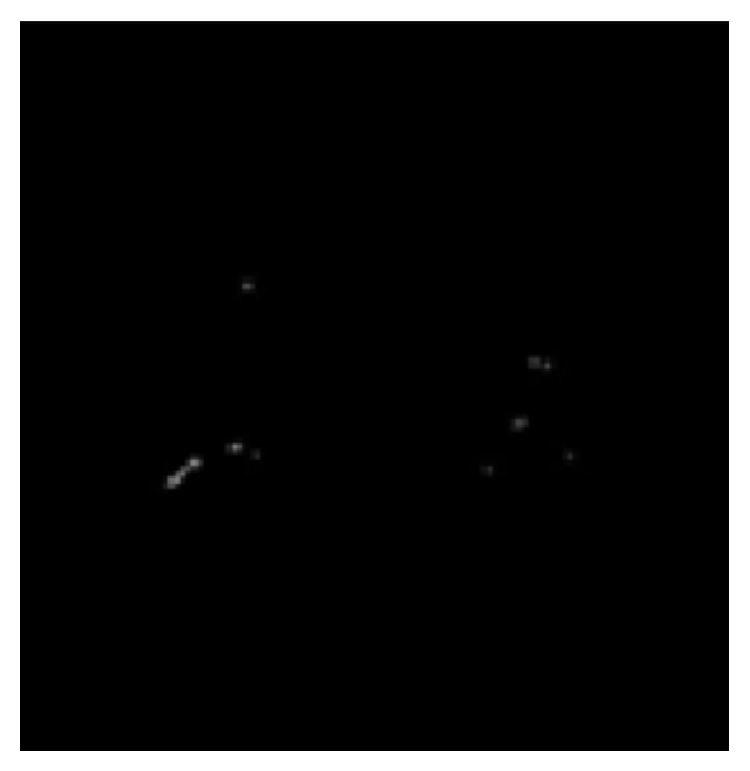
Pulmonary nodule.

Shape of the nodules is a “cap” class object. High value of mean shape index value means more sphericity of the object and so it is the indication of nodules. We defined shape index value “0.76” as a threshold value. All objects having shape index values greater than 0.76 are nodules. All objects having mean index value less than or equal to 0.76 have been deleted from the slice image in order to show only the nodule objects. These objects were vessels, airways, or other structures. Identified pulmonary nodules (PNs) are shown in Figure 5.

## 3. Results and Discussion

**Figure 6:**
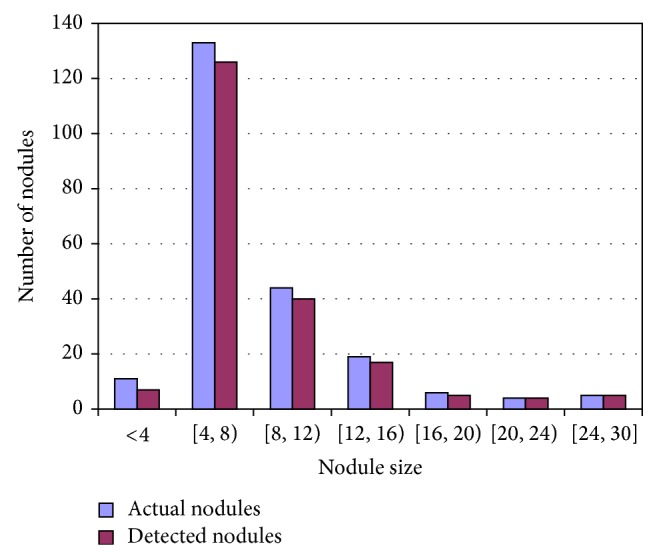
Number of nodules detected.

Results are the worth of the research. Testing is important for verification of the results. We used lung CT scan of LIDC-IDRI database [24] for experimentation and evaluation. The description and selection criterion of the images are stated in Section 2.1. We achieved 92% sensitivity with full automation taking nodule size 3–30 mm. The number of actual nodules and nodules detected is shown on bar graph in Figure 6. The graph shows that the number of nodules is large when the size of the nodules is less than 10 mm. The detection rate is high when the nodule size is large.

The proposed system was compared with existing methods on lung nodule detection. The parameters of the comparison are Sensitivity, False Positives (FPs), and Nodule Size.  Table 1 shows the comparison of the proposed method. Making an exact comparison is difficult as some researchers did not use standard dataset for evaluation of their method or they used different evaluation parameters. However, mentioned parameters together provide a reliable comparison for the proposed method.

As manifested from Table 1, the proposed method reflects better sensitivity than the mentioned contemporary methods. Some researchers did not report nodule size. We evaluated nodules of all sizes 3–30 mm. Dehmeshki et al. [13] showed 84% sensitivity with the fully automated system and 100% sensitivity with manual intervention. We achieved 92% sensitivity with complete automation. Sensitivity of Dehmeshki et al. [13] with manual intervention is better. However, manual intervention has its own problems and difficulties which may set aside higher sensitivity benefit. Moreover, Dehmeshki et al. [13] did not mention nodule size and number of false positives. Both these parameters influence the sensitivity.

We explored the nodule size and sensitivity calculus. Nodule detection is a function of variable threshold levels, shape index threshold, and nodule size criterion. Shape index threshold was found empirically. Reason for the better results of the proposed method as compared to the contemporary research works (as shown in Table 1) is better detection of low intensity nodules and nodules attached with other pulmonary structures.

## 4. Conclusion

Accurate lung nodule detection is vital for the diagnosis of lung cancer. We proposed a novel automated lung nodule detection system. Comprehensive testing revealed that the proposed threshold based method provides encouraging results in detecting pulmonary nodules on lung CT slice images. Shape index threshold has proved good for false positive reduction. It helped remove most of the tissues such as apical scarring, blood vessels components, and some objects which resulted from partial volume effect. In the future, we would characterize the nodules and measure the volume of the malignant nodules.

## Figures and Tables

**Algorithm 1:**
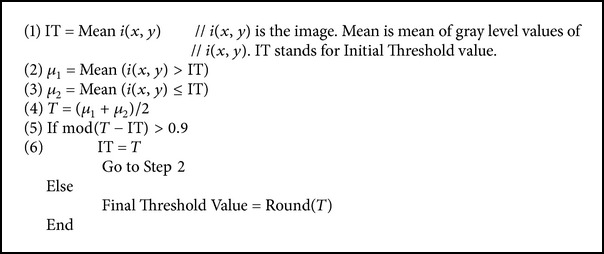


**Table 1:** Comparison.

Sr. number	Study	Year	Nodule size (mm)	Sensitivity (%)	FPs
1	Dehmeshki et al. [13]	2008	— —	84 100	— —
2	Ye et al. [25]	2009	3–20	90.2	8.2
3	Messay et al. [26]	2010	3–30	82.66	3
4	Tan et al. [27]	2011	—	87.5	4
5	Cascio et al. [28]	2012	—	88	2.5
6	El-Baz et al. [29]	2013	—	82.30	12
7	Proposed method		3–30	92	6
